# Challenges in quantifying the patient-reported burden of herpes zoster and post-herpetic neuralgia in the UK: learnings from the Zoster Quality of Life (ZQOL) study

**DOI:** 10.1186/1756-0500-6-486

**Published:** 2013-11-25

**Authors:** Stuart Carroll, Adam Gater, Linda Abetz-Webb, Fiona Smith, Dirk Demuth, Azharul Mannan

**Affiliations:** 1 Sanofi-Pasteur MSD, Berkshire, UK; 2 Adelphi Values, Cheshire, UK; 3 Adelphi Real World, Cheshire, UK

**Keywords:** Herpes zoster, Post-herpetic neuralgia, Real-world, Burden

## Abstract

**Background:**

Acute presentation of herpes zoster (HZ) and the subsequent development of post-herpetic neuralgia (PHN) can have a significant impact on patients’ lives. To date, evidence regarding the human and economic burden of HZ and PHN in the UK is limited. To address this knowledge gap a national, multicentre, large-scale real-world study was conducted to inform the scientific community and healthcare decision-makers. This paper outlines difficulties encountered and challenges to conducting real-world studies in the UK, methods used to overcome these hurdles and strategies that can be employed to promote and facilitate the conduct of future studies.

**Findings:**

The Zoster Quality of Life (ZQOL) study is the first UK-wide and largest observational study investigating patient burden associated with HZ and PHN. A total of 383 patients (229 HZ; 154 PHN) over the age of 50 years were recruited from 42 primary and secondary/tertiary care centres. Patient-reported outcome (PRO) assessments of pain, quality of life and treatment satisfaction were completed by all participants and supplemented by clinical information from participating physicians.

Key challenges encountered during the conduct of this study can be broadly categorised as follows: 1) identification of centres willing/able to participate in the study: lack of resources and limited research experience were major barriers to recruitment of centres for participation in the study; 2) obtaining local research & development (R&D) approval: lack of clearly defined processes and requirements specific to real-world studies and limited degree of standardisation between R&D departments in approval procedures led to significant variability in submission requirements and lead times for obtaining approval; 3) recruitment of study participants: rates of recruitment were slower than anticipated, meaning it was necessary to extend the study recruitment period and increase the number of participating centres.

**Discussion:**

Initiatives designed to promote and facilitate the conduct of research in the UK are important for real-world studies. The ZQOL study shows that opportunities exist for real-word research. However, streamlining the R&D approval process where possible and further incentivising the participation of primary care centres in such studies would help to further facilitate the generation of real-world evidence to inform healthcare decisions.

## Background

Herpes Zoster (HZ), often referred to as ‘shingles’, is a viral condition resulting from reactivation of latent varicella-zoster virus (VZV) which is responsible for childhood ‘chickenpox’. The condition is characterised by a painful skin rash and blisters on one side of the body. It is estimated to affect one in four persons during their lifetime, with an estimated 200,000 episodes in the UK annually [1-3]. Whilst anyone previously infected with the VZV (i.e. has had “chickenpox”) is at risk of developing shingles, incidence increases with age and is most prevalent among those patients aged 50 years and over [4]. The dermatological rash and pain associated with HZ typically resolve within one month of presentation, however approximately 20% of sufferers will continue to experience pain in the area of the rash, following resolution [4]. Although variable definitions exist, instances where pain persists for > 90 days after the onset of the rash are typically referred to as post-herpetic neuralgia (PHN) [5]. The incidence of PHN increases markedly with age and is also associated with the severity of pain experienced during the preceding HZ episode as well as the extent of the rash [6].

Previous cross-sectional, epidemiological studies have demonstrated that the pain associated with HZ and PHN can have a significant impact on patients’ Health-related Quality of Life (HRQoL) [6-13]. In particular, patients who develop PHN may experience pain and associated reductions in HRQoL long after the initial acute presentation of the HZ rash, with some patients reporting persistent pain for more than ten years [10]. In addition to the burden encountered by the individual, HZ and PHN are also associated with considerable societal burden. Care provision for HZ and PHN patients, in terms of visits to primary care (general practitioner centres) and outpatient secondary/tertiary care centres (specialist pain clinics and ophthalmologists), inpatient visits (hospitalisations) and prescription costs, for example, is at considerable cost to healthcare systems [14-19]. In addition, HZ and PHN are also associated with significant indirect costs, primarily in terms of loss of productivity for patients and caregivers [9,15]. This is particularly relevant for those patients with Herpes Zoster Ophthalmicus (HZO) and/or with long-standing or severe PHN.

Key to worldwide health authority initiatives is the prioritisation of preventative measures, so as to reduce the burden on healthcare conditions on individuals, healthcare systems and society as a whole. This is evident in the UK by the recent introduction of the Quality, Innovation, Productivity and Prevention (QIPP) initiative, which outlines the UK Government’s commitment to supporting research to provide new knowledge needed to improve health outcomes and reduce inequalities by facilitating advances in disease prevention, diagnosis and treatment [http://www.improvement.nhs.uk/Default.aspx?alias=www.improvement.nhs.uk/qipp; Accessed 13th August 2012]. Typically, management of HZ/PHN has centred upon the use of antivirals (usually prescribed within 72 hours of the onset of the rash) to treat the initial infection and initial regulation of pain using prescribed and/or over-the-counter pain medication and prescribed antidepressants and anticonvulsants thereafter to modulate persisting pain. Recent years, however, have seen documented evidence of the efficacy of VZV vaccines in the prevention of HZ and PHN episodes and attenuation of the severity of HZ and PHN episodes and associated impact [20].

The value of real-world data summarising patients’ experiences of living with a health condition in informing decisions regarding access to new medicines in national markets is becoming increasingly recognised. Indeed, as stipulated in the Government’s Health and Social Care Bill (HSCB), the proposed commissioning outcomes framework, which is a fundamental foundation of the proposed NHS reforms in England, will place a strong emphasis on promoting decision-making that is driven by the patient experience – as likely measured by patient reported outcome (PRO) measures [21].

To date, there has only been limited real-world evaluation of the burden of HZ and PHN specific to UK patients, and what research has been conducted is limited by relatively small sample sizes and a lack of geographic representation [9]. However, UK-specific data is valuable to generate best available evidence for appraising the cost-effectiveness of strategies for the prevention and/or management of HZ and PHN (including VZV vaccines). So far, such appraisals have incorporated data derived from HZ and PHN patients in the US and Europe [14,22]. There is a need, therefore, for the generation of more information to accurately ascertain disease burden in UK patients, a view expressed by the UK Joint Committee on Vaccination and Immunisation (JCVI) [23,24]. To address this need the Zoster Quality of Life (ZQOL) study, the first UK-wide and largest evaluation of the experiences of HZ and PHN patients conducted to date, was initiated in August 2009.

The current paper outlines experiences and insights from the conduct of the ZQOL study with a reflection on the challenges and opportunities for the conduct of real-world research in the UK.

## Methods

**Figure 1:**
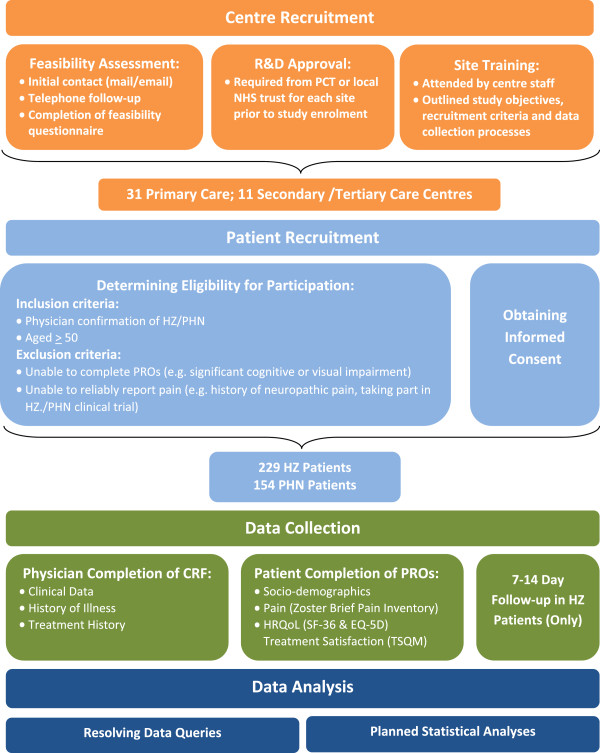
Overview of ZQOL study methodology.

The ZQOL study was designed to provide an in-depth evaluation of the clinical presentation and management of HZ and PHN and the burden incurred by patients with these conditions. Data was collected from patients and treating physicians using a combination of validated PRO measures and specially designed questionnaires. An overview of the ZQOL study methodology and processes are provided in Figure 1.

### Clinician recruitment

The management of HZ and PHN is largely based in primary care, with referrals to secondary/tertiary care usually reserved only for those patients with HZO or persistent or severe PHN (ophthalmologists and specialist pain clinics, respectively) [25]. As such, patients were to be recruited to the ZQOL study largely by primary care centres, with a smaller proportion of patients (predominately PHN) recruited by secondary/tertiary care specialist pain clinics and ophthalmologists.

### Patient recruitment

Patients presenting to doctors with a visible HZ rash (i.e. unilateral crop of blisters and scabs in a dermatomal distribution) were eligible for participation. Diagnosis of HZ was not confirmed by polymerase chain reaction (PCR) and this could be a considered a potential limitation of the study. However, the distinctive appearance and distribution of the HZ rash (a unilateral crop of blisters and scabs in a dermatomal distribution) mean that diagnosis of HZ is usually clear [26]. In addition PCR assessments are fraught with logistical difficulties in primary care and so are rarely used in practice; [27] therefore, the current method of diagnosis is reflective of that used in ‘real world’ practice. Additional inclusion criteria of note was the focus only upon adults over the age of 50, in accordance with data suggesting that the incidence and severity of HZ increases significantly in this age range [4].

To promote the ecological validity of the study, extensive exclusion criteria (as would typically be adopted in a clinical trial) were not employed. However, to protect the integrity of study findings, HZ patients with health impairments that may make it difficult for them to complete the required battery of PRO instruments (e.g. those deficits in cognitive functioning or visual impairments) or those whose medical background may make it difficult to interpret study results (e.g. patients who have taken part in a clinical trial related to HZ, pain and/or immunomodulating therapy in the past 6 months or patients who were previously experiencing neuropathic pain in the dermatomal region of their HZ rash prior to the onset of HZ) were excluded from participation.

### Data collection

At the initial site visit, once informed consent had been obtained and participant eligibility for participation confirmed, patients were asked by study staff to complete a combination of validated PRO questionnaires (including the Zoster Brief Pain Inventory (ZBPI) [28], MOS 36-item Short-Form Health Survey (SF-36) [29], EuroQoL 5 Dimensions questionnaire (EQ-5D) [30] and the Treatment Satisfaction Questionnaire for Medication (TSQM) Version II [31] and those specifically designed for the purposes of the study (i.e. patient socio-demographic and clinical history questionnaire) All questionnaires were bound in a study booklet so to ensure standardisation of presentation and order of completion among all patients.

There is no consensus in the research literature as to the stage of the acute HZ episode at which pain and HRQoL impact are at their highest [7,32]. Therefore, to ensure that the patient-reported impact of acute HZ was not underestimated by data collection on initial presentation, all HZ patients were provided with additional copies of the validated PRO questionnaires which they were asked to complete at home and return 7–14 days after their original visit.

Clinical data and details of treatment history were collected by the recruiting doctor from patient consultation and medical records, and recorded using a study-specific case report form (CRF). The CRF ensured that HZ complications other than PHN and HZO (e.g. Delayed contralateral hemiparesis, Ramsay Hunt syndrome and zoster sine herpete) could be recorded; however evidence shows that these are very rare [33].

### Planned analyses

Data from the ZQOL study is to be analysed in accordance with a formal Statistical Analysis Plan (SAP) developed a priori. All study data is subject to extensive quality control checks and queries for doctor-reported information is to be resolved where possible. Specific analyses are designed to quantify the burden associated with HZ and PHN by comparing mean scores on generic quality of life questionnaires (e.g. SF-36 and EQ-5D) derived from ZQOL study participants with published aged-matched norms derived from the general population [34,35]. In addition, analyses design to test hypothesised differences in outcomes by variables including age, gender, antiviral use and pain severity are planned, Similarly, analyses designed to explore potential predictors of pain and HRQOL are also planned.

## Findings

A number of key challenges were encountered during the conduct of the ZQOL study, which presented some hurdles to the collection of data from a sufficient number of patients within the pre-determined time-frame. Some of the challenges encountered were a consequence of organisational factors within the NHS including: 1) identification of centres willing/able to participate in the study; 2) enrolling centres into the study (incl. obtaining overall NRES approval for the study and R&D approval for individual centres); and 3) recruitment of study participants and data collection. Further details of these challenges and strategies for overcoming or minimising the impact of these are discussed (a summary is provided in Table 1).

**Table 1:** Overview of ZQOL study challenges, solutions and implications

**Key challenges**	**Solutions**	**Future implications**
**1) Identification of centres willing/able to participate in the study**		
• Identifying centres with interest/capacity to participate in research and the correct people to discuss participation with proved difficult via conventional means (unsolicited mail/email). • Limitations in dedicating time or resources to external research projects due to clinical workloads and lack of designated support staff were common reasons for non-participation cited by centres. Levels of remuneration provided to centres for participation in the study were considered insufficient by some centres. • Availability of NHS staff participating in research having completed Good Clinical Practice (GCP) training.	• The ZQOL study was accepted into the National Institute for Health Research Clinical Research Network (NIHR CRN) portfolio. NIHR were able to offer wide range of support services including: o Access to existing networks of centres with interests in participating in research. o Provision of support to centres interesting in participating in research. o Exploring centre eligibility for additional remuneration for participation in NIHR CRN-approved studies. o Delivering GCP training to centres interesting in participating in studies.	• Experiences indicate the need for greater links between commissioners of research and primary/secondary care centres. • A refocusing of targets for primary care centres, to provide staff with opportunities and incentives for partaking in research is needed. • Current R&D approval process makes no concessions for non-interventional research. R&D requirements should be proportionate to the risks associated with patient participation in such studies.
**2) Obtaining Ethics and Research & Development (R&D) Management Approval**		
• Information and documentation required to support R&D applications was a barrier to participation for a number of centres. • Significant variation in timelines for R&D approval at a local and regional level (i.e. England, Scotland, Wales & Northern Ireland) led to significant delays in the study. • R&D approval process requires that extensive tri-partite agreements between the study sponsor, co-ordinating CRO and R&D department be agreed and signed. However, there is no standard template available for such agreements.	• ZQOL study organisers implemented a number of practical solutions including: o Minimising the number of R&D approvals by seeking participation of clusters of centres in the same NHS trust. o Implemented a staged study roll-out such that R&D applications for key centres were prioritised and R&D applications could occur in parallel to patient recruitment. • NIHR CRN can also provide assistance: o As a NIHR-CRN portfolio adopted study, more likely to be considered for priority review by R&D depts. o NIHR CRN local research network teams are able to offer support and guidance to co-ordinating CROs throughout England.	• Current R&D approval process makes no concessions for non-interventional research. R&D requirements should be proportionate to the risks associated with patient participation in such studies. • A move to standardise R&D approval process across NHS trusts in England would reduce burden (and barriers) to research for study organisers and NHS staff interested in participating in research.
**3) Recruitment of Study Participants**		
• Recruitment of HZ patients in primary care was slower than envisaged, a likely result of practical and organisational factors: o As an acute condition, HZ patients had to be recruited on initial presentation to physicians and did not allow searches of eligible participants via patient medical records. o At the time of the study there will only limited opportunities for notifications and reminders to be incorporated into electronic-record systems on presentation of incident cases. • Lack of unique identifiers or codes in electronic-record systems made it difficult to identify PHN patients for participation in the ZQOL study.	• To ensure that study recruitment quotas were met, additional centres were recruited for participation in the study and study timelines were extended. • NIHR CRN local research networks and the co-ordinating CRO were in regular contact with centre staff so to ensure to provide support where needed.	• Standardisation of medical record keeping and greater integration of record and monitoring systems would be to the benefit of facilitating real-world research. • Centres having systems in place to confirm the feasibility of recruitment numbers and having opportunity to work alongside study organisers could facilitate the development of study inclusion/exclusion criteria that are less restrictive to recruitment in real-world practice.

### Identification of centres willing/able to participate in the study

Difficulties in the initial recruitment and retention of primary care centres for voluntary participation in research studies are widely reported [36]. Furthermore, similar hurdles have also been noted in prior UK investigations of the burden of HZ and PHN. Scott et al. (2006) [9], for example, approached 45 primary care centres for participation in a cross-sectional study, only 18 of which agreed to do so [9]. This is despite the fact that all invited centres had either participated in a previous shingles research study [20], were associated with the Academic Department of General Practice or had attended an educational meeting about HZ. It is reasonable to question, therefore, whether receptivity and actual rates of participation in such a study would be lower among a wider sample of community primary care centres with arguably less motivation or vested interest for participation.

Experiences during the recruitment of centres for the ZQOL study were indeed reflective of the above assertion, as inherent difficulties in identifying centres with an interest in participating in this study and research in general were noted. This was particularly evident during the initial stages of the project at which point a large number of potential centres for participation were identified through the use of specialist databases (e.g. http://www.specialistinfo.com). However, unsolicited contact with these centres via conventional means (e.g. telephone/email/fax) proved relatively unsuccessful in identifying centres willing to participate, with only a small number of centres recruited as a result of such efforts.

These experiences highlight the challenges for researchers in identifying primary and secondary/tertiary care centres willing to assist in the conduct of research. In a drive to promote the conduct of research in the UK, in 2006 the UK Department of Health (DH) set up The National Institute for Health Research (NIHR). A key component of this organisation is the NIHR Clinical Research Network (NIHR CRN), designed to provide an infrastructure by which to facilitate the conduct of high-quality studies within the NHS. One of the key objectives of the NIHR CRN is ‘being able to direct researchers towards local patient populations and research capacity, so that participant targets can be achieved’. At the heart of NIHR CRN’s activities is the NIHR CRN Portfolio which consists of high quality research projects that are eligible for support and can benefit from the infra-structure of the CRN. Whist comprising mainly randomised control trials (RCTs), the portfolio also supports other types of well-designed research. Once the ZQOL study had been accepted into the NIHR CRN portfolio, the NIHR CRN played an instrumental role in the identification of centres that would be willing to participate in the study. The avenue through which potential participating centres are approached and invited to participate in the research studies has been identified as a successful recruitment of centres for participation in such studies [37]. Using the support of NIHR CRN staff in this capacity ensured that the correct personnel (or ‘gatekeepers’) at potential participating centres were contacted in relation to this study. There can often be a degree of scepticism and wariness among NHS centres as to the role of the pharmaceuticals industry in sponsoring real-word research. That the ZQOL study was an approved CRN portfolio study added an additional degree of scientific credibility to the study, and was cited by many centres as one of the reasons that they agreed to participate in the study. Indeed, our experiences suggest that many centres, particularly in primary care, are only interested in participating in CRN portfolio adopted studies.

However, even with the assistance of NIHR CRN, experiences during the centre recruitment process highlighted some barriers to centre willingness and eligibility for participation in the study. One of the most frequently cited barriers to participation, even among centres showing initial interest in participating in the study, was a lack of capacity to dedicate time or resources to external research projects due to their already considerable work commitments. This was particularly evident among primary care centres; many of whom did not have designated support staff that were able to assist with research activities, an assertion consistent with previous research which has identified time and work commitments as a major barrier to participation in primary care research [38]. Primary care workloads are also very much influenced by seasonal variations in illness. At the point at which centres were being contacted regarding participation in the ZQOL study, for example, the swine flu pandemic had hit the UK and primary care centres were at the forefront of vaccination delivery programmes among patients most at risk of flu. It is likely therefore that these extra demands on primary care centres may have influenced decisions to decline participation in the current study. Finally, as a further reflection of time being a premium resource within primary and secondary/tertiary care centres, a number of centres asserted that the financial remuneration (paid on the basis of per-patient enrolled as is typical for these types of studies) offered for participation in the study was not enough to justify the time that would need to be spent on the project. Rates of remuneration for participating centres, however, were in accordance with UK ethics and industry-specific guidelines for payments to healthcare professionals.

As noted, the assistance of the NIHR CRN proved invaluable in identifying centres with a strong interest in such research. In addition, the NIHR CRN provides support to centres looking to participate in research to reduce the anticipated burden that may be incurred by site staff for taking part in such a study. There is also an appreciation within the NIHR CRN of the remuneration limits that may be stipulated by ethics committees for participation in industry-led research. Sites participating in NIHR CRN approved studies, therefore, are eligible for additional remuneration, provided by the research network to facilitate and promote participation in such studies.

Current guidelines among NHS trusts require that all researchers and centre personnel involved in clinical research with human subjects demonstrate evidence that they have undertaken Good Clinical Practice (GCP) training. Compliance with GCP ensures that the rights, safety and well-being of research subjects are protected in accordance with the principles of the Declaration of Helsinki and other internationally recognised ethical guidelines. Despite the non-interventional nature of the ZQOL study, R&D departments were insistent that evidence of up-to-date GCP training be provided before approving centres for participation in the study. However, as noted many of the centres contacted for potential participation in the ZQOL study had only limited research experience and a vast proportion had not completed GCP training. It was therefore necessary for centre-staff to complete accredited-GCP training courses, in order to fulfil this requirement, before being considered for participation.

As part of their role in the facilitation of a portfolio adopted study, therefore, NIHR CRN staff were able to deliver GCP training to staff at centres interested in participating in the ZQOL study. As the content of the GCP training is very much tailored towards clinical research, it was also a requirement that all centres take part in a ZQOL study-specific training session, led by the co-ordinating contract research organisation (CRO), in which key requirements for real-world studies and this particular study were outlined. In addition, throughout the course of the study, centres had access to a comprehensive support system including NIHR CRN staff and those from the co-ordinating CRO.

### Obtaining ethics and research & development (R&D) management approval

In accordance with current research governance frameworks in the UK, any research involving National Health Service (NHS) patients must be approved by the National Research Ethics Service (NRES) to assure that *‘any anticipated risks, burdens or intrusions will be minimised for the people taking part in the research and are justified by the expected benefits for the participants or for science and society’*[39]. While the ethical approval process for the conduct of research in the UK is robust, in the past it has been criticised as a significant barrier to the conduct of academic and industry sponsored research [40-45]. These challenges were particularly evident for multicentre studies, where there was a requirement for independent consideration of studies at a national and local level. The lack of standardised systems and processes often resulted in considerable administrative burden and delays in attaining notice of approval.

In recent years, attempts have been made to streamline the ethical review process for research involving NHS participants. For example, changes in the NRES now mean that it is no longer necessary for studies to be considered and approved at both a national and a local level. Furthermore the introduction of the on-line Integrated Research Application System (IRAS) has also sought to minimise the administrative burden associated with NRES submissions such that all information required for study review can be submitted via standardised and centralised means. In standardising these procedures researchers are informed that they can now expect an opinion on their study to be received within 60 days of receipt of application by the main REC. Indeed, NRES approval for the ZQOL study was obtained within this timeframe.

However, in addition to obtaining NRES approval, it is also a requirement under the 2005 Research Governance Framework for Health and Social Care, that any research that uses NHS patients, staff, premises or resources also obtain formal approval from the Research & Development (R&D) departments of each of the local NHS organisations in which research is to take place, before research can commence at the respective centres. Without this approval indemnity/insurance cannot be assumed to cover the proposed research activity.

Coordinating R&D management approval for the ZQOL study proved to be a challenging and time consuming process for the co-ordinating CRO and participating centres. There are currently no concessions in place within the R&D approval process for non-interventional research such that the requirements and requested level of supporting documentation are equivalent to that expected for a clinical trial intervention research project. Following completion of the main NHS R&D form by the chief investigator, therefore, there was wealth of information and evidence to be provided by the principal investigator at each of the participating centres to fulfil R&D requirements. This included completion of a Site-Specific Information Form (SSI) to be submitted electronically via the IRAS system alongside evidence of GCP training and up-to-date Curriculum Vitaes (CVs) for all staff involved in the project. As a result of the demands of the R&D approval process, a number of centres who had originally agreed to participate in the study withdrew from participation.

Despite recent attempts to standardise the NRES and R&D application process via introduction of the IRAS system, experiences during the ZQOL study highlight a distinct lack of standardisation in R&D approval requirements and processes. Firstly, unlike NRES applications for which there is a designated 60-day review policy, there are no fixed timelines for R&D. Applications for R&D approval can be made in parallel with NRES submissions but experiences from the ZQOL study highlighted wide variation at a local and regional level (i.e. England, Scotland, Wales and Northern Ireland), with approval being obtaining in as little as 3–4 days or as much as 152 days from submission of R&D application. The average time from R&D submission to approval across sites enrolled in the study was 45 days. As part of the R&D approval process it was also necessary for extensive tri-partite agreements to be agreed and signed by the study sponsor, co-ordinating CRO and R&D department. There currently exists no standardised template specifically designed for non-interventional research, such that the time taken to negotiate these agreements contributed to overall approval timelines.

The difficulties faced by study organisers in terms of obtaining R&D approval is recognised by the NIHR CRN whose local research network teams are able to offer support and guidance to co-ordinating CROs and participating centres throughout England. Furthermore, as an NIHR-CRN portfolio-adopted study the ZQOL study is also likely to have been considered for priority review by R&D departments. Indeed, studies now adopted into the NIHR-CRN portfolio can also take advantage of the NIHR co-ordinated system for gaining NHS permission. In addition to the support provided by NIHR CRN, there are a number of practical solutions that can be adopted to minimise the burden of R&D submissions for multi-centre studies. Firstly, while it was important to ensure adequate geographical distribution of centres throughout the UK, it was possible to minimise the administrative burden of R&D submissions by seeking participation of clusters of primary care centres within the same NHS trust. Secondly, a staged study roll-out meant that once approved by NRES, R&D approvals for pending study centres could be on-going whilst the study was in field.

### Recruitment of study participants

Sample size calculations are important in observational research to ensure that studies possesses adequate statistical power to allow accurate, reliable and valid conclusions to be drawn from study data [46]. However, failure to reach designated recruitment targets is a commonly reported problem in primary care research with less than 33% of studies meeting their recruitment target and approximately 45% recruiting less than 80% of their original target [47]. Consistent with this assertion, difficulties recruiting HZ patients in the UK have been reported previously. Scott et al. (2006) [9], for example, estimated that a population of 150,000 served by their study centres would identify 200–300 cases of HZ and 45 cases of PHN in 8 months [9]. In reality, however, only 96 patients were referred to the study, of which 70 enrolled and only 65 completed, highlighting the fact that once centres are enrolled, challenges still exist in terms of recruiting study participants. This raises the question as to whether there is a realistic understanding and appreciation of the practical challenge pertaining to recruitment. If centres could utilise a system of validation to confirm the feasibility of recruitment numbers and work with study organisers to develop inclusion/exclusion criteria that foster a more dynamic approach to research, improvements could be realised.

Prior to study start-up a small scale feasibility study was conducted, the findings of which estimated lead times for patient recruitment to be 6–8 months, with each centre expected to recruit approximately 7 HZ and 5 PHN patients over this period. Contrary to expectations, interim recruitment rates for the ZQOL study revealed slower recruitment rates than originally envisaged, particularly among primary care centres. Furthermore, despite going through the hurdle of the R&D process, 8 participating centres did not contribute any data to the study. There are a number of factors thought to have contributed to the slow recruitment rate within such centres. Firstly, this may be explained by constraints in terms of resources for obtaining patient and physician completed information. Secondly, in accordance with the typical management of HZ and PHN, specified recruitment targets assumed that the majority of HZ patients would be recruited from primary care. As an acute disease, primary care centres were to recruit patients with incident cases of HZ on initial presentation to staff at participating centres. As such, there was no opportunity to utilise centre records or databases to identify existing cohorts of patients who could be invited to participate in the study. Furthermore, at the time of the study there was only limited opportunity for study notifications and reminders to be incorporated within electronic-record systems used by centres (e.g. EMIS, iSOFT) on presentation of incident cases. In addition, it became apparent, during the course of the study, that there was little standardisation in record-keeping practices in terms of systems used (e.g. EMIS, iSOFT) and information recorded by centres. In many cases, for example, there was no unique identifier or code in place to identify patients who may be experiencing PHN, offering support to prior assertions that as many as 80 % of patients with PHN may not have this diagnosis specified within administrative systems [16]. Acknowledging this coding issue, it is important to consider alternative strategies to identify eligible participants.

Finally, it is worth noting that remuneration for patients was not identified as a significant barrier to recruitment to the ZQOL study. Patient incentives were designed to cover estimated out-of-pocket expenses for participation in the study only (e.g. travel to and from the study site). This ensured that, in accordance with ethical standards, informed consent to participate in the study was free from coercion and undue influence that may occur when incentives offered to research participants are in excess of the level of compensation expected.

As is a common consequence in such studies [36], due to the experience of such difficulties and to ensure that recruitment targets were met, study timelines were extended. To account for centres where recruitment was proving particularly difficult, additional centres were also enrolled to the study, where possible within the same NHS trust so to minimise the burden of the R&D approval process. The help of the NIHR CRN local research teams was also important for liaising with staff at participating centres as well as the co-ordinating CRO to address any difficulties encountered and provide assistance where needed. In addition to maintain motivation and investment in the study among participating centres, centre staff were routinely informed of the status of the project via regular project update calls. In addition, more formal written updates (e.g. end of year update memos, newsletters, etc.) were sent to all participating centres.

In light of these initiatives, pre-specified recruitment targets for the ZQOL study were eventually met. The final list of participating study sites represented a diverse spread of primary and secondary care centres throughout the UK, addressing some of the concerns with existing research conducted in the UK thus far.

## Discussion

Recent years have seen a decline in the conduct of clinical trial research in the UK [48]. However, a key feature of the UK Government’s reforms to the NHS is the promotion of the conduct of research “to improve health outcomes and reduce inequalities” [21]. One way to ensure continued growth in the UK research arena, as recognised by the Association of the British Pharmaceutical Industry (ABPI), is to promote the conduct of ‘real-world’ research in a UK context [49]. Whilst randomised control trials (RCTs) are able to provide evidence of the efficacy and safety of medical interventions, real-world studies provide important generalisable evidence of the ‘effectiveness’ and value of such interventions in normal clinical practice. The value of such evidence is being increasingly recognised by regulatory authorities; in particular the generation of robust real-world evidence will be key to the Value-Based Pricing scheme applied to medicines, which is set to be introduced to the NHS from 2014 [50]. Real-world data concerning current burden of disease and unmet medical need will be important for informing economic and budget impact models used to determine cost-effectiveness thresholds for pricing and reimbursement.

As the largest and first UK-wide investigation conducted within HZ and PHN patients, the ZQOL study was designed to provide much needed UK-specific information on the impact of HZ and PHN and their management, from both a human and wider economic perspective. However, as highlighted challenges and hurdles were encountered during the course of this study, which could have prevented achievement of participant recruitment targets (essential to ensure the statistical power of the study) within the pre-determined time frame. Some of the contributory factors are arguably reflective of organisational, structural and cultural characteristics of the NHS and its associated organisations.

In recognition of some of these challenges, support networks and modification to NHS systems have been put into place. As noted, for example, local research networks provided by the NIHR CRN are able to guide and support research co-ordinators right throughout the research process. The introduction of the IRAS system has also sought to streamline and reduce the administrative burden of the NRES and R&D approval process. However, whilst recognising the need to assure scientific quality and ethical standards in real-world studies, current systems and requirements are considerable hindrance to the conduct of such studies. At present, for example, current regulatory and governance frameworks do not distinguish ‘non-interventional’ real-world studies from ‘interventional’ RCTs. As such, many of the requirements imposed on real-world studies (e.g. need for all participating NHS staff to have GCP training), seem disproportionate to the risks associated with such studies. Streamlining and standardising this process, therefore, would go a great way to minimising the barriers to the conduct of such studies.

As highlighted during the conduct of the current study, it is important to note that should the UK Government wish to promote health research in the UK, then such research needs to become engrained within the culture of the NHS. For primary care centres, in particular, a lack of capacity for research in the context of current workloads and limited research experience was evident. A refocusing of targets for primary care centres to provide staff with opportunities and incentives for partaking in research is therefore needed.

Whilst the current paper has focussed specifically on challenges to the conduct of real-world studies in the UK, it is important to appreciate that similar difficulties also face researchers in other countries throughout Europe. However, increasing recognition and appreciation of these difficulties by UK authorities (such as the ABPI) is designed to promote the adaptation of current systems and process in order to facilitate the conduct of such studies. This is especially important as the need for information derived from real-world studies grows ever greater. In considering the implementation of these changes, the UK can be positioned at the forefront of health research.

## Conclusions

The value of ‘real-world’ research is being increasingly recognised by healthcare decision makers. The ZQOL study demonstrates that opportunities exist for real-word research in the UK, but also highlights significant challenges that may present for researchers wishing to conduct such research in the UK. These experiences suggest that initiatives designed to promote and enable the involvement of healthcare professionals in research, streamline the R&D approval process and assist with the identification of eligible participants would help to further facilitate the generation of real-world evidence in the UK to inform healthcare decisions for UK patients.

## Competing interests

AG/LA and FS/DD are employees of Adelphi Values and Adelphi Real World, respectively; health outcomes agencies commissioned by Sanofi Pasteur MSD, to conduct, analyse and communicate findings from this research on their behalf. SC and AM are employees of Sanofi Pasteur MSD, a provider of a herpes zoster vaccine approved in the European Union. All authors have not further competing interests to declare.

## Authors’ contributions

AG, LA, FS and DD were responsible for the design and conduct of the study. SC & AM facilitated the conduct of the study. AG, SC and AM drafted the manuscript. All authors read and approved the final manuscript.
